# International Federation of Clinical Chemistry (IFCC): Scientific Division, Committee on pH, Blood Gases and Electrolytes: *Guidelines for Transcutaneous*po_2_*and*pco_2_ *Measurement*

**DOI:** 10.1155/S1463924689000465

**Published:** 1989

**Authors:** P. D. Wimberley, R. W. Burnett, A. K. Covington, A. H. J. Maas, O. Mueller-Plathe, O. Siggaard-Andersen, H. F. Weisberg, W. G. Zijstra

**Affiliations:** 1 Department of Clinical Chemistry Herning Hospital Herning DK 7400 Denmark

## Abstract

This document provides guidelines for the terminology, methodology, and for the interpretation of data obtained from the use of skin (transcutaneous) po_2_ and pco_2_ electrodes. The transcutaneous technique has found special application in newborn infants. The causes of analytical bias with respect to arterial blood gas values, and imprecision obtained with transcutaneous pco_2_ electrodes, are reviewed. Electrode temperatures above 44°C should not be used routinely, and, at a measuring temperature of 44°C, the measuring site should be changed at least every 4 h to avoid skin burns.


Journal of Automatic Chemistry Vol. 11, No. 5 (September–October 1989), pp. 235–239

### International Federation of Clinical Chemistry (IFCC): Scientific Division, Committee on pH, Blood Gases and Electrolytes: *Guidelines for Transcutaneous $p_{O_2}$ and $p_{CO_2}$ Measurement*†

**P. D. Wimberley (DK), R. W. Burnett (US), A. K. Covington (UK), A. H. J. Maas (NL), O. Mueller-Plathe (FRG), O. Siggaard-Andersen (DK), H. F. Weisberg (US) and W. G. Zijstra (NL)**

*This document provides guidelines for the terminology, methodology, and for the interpretation of data obtained from the use of skin (transcutaneous) $p_{O_2}$ and $p_{CO_2}$ electrodes. The transcutaneous technique has found special application in newborn infants. The causes of analytical bias with respect to arterial blood gas values, and imprecision obtained with transcutaneous $p_{CO_2}$ electrodes, are reviewed. Electrode temperatures above 44 °C should not be used routinely, and, at a measuring temperature of 44 °C, the measuring site should be changed at least every 4 h to avoid skin burns.*

*Keywords: Electrodes; Blood gases;* In vivo *measurements; Heated skin.*

### 1. Introduction

Electrodes for transcutaneous measurements of oxygen and carbon dioxide are widely used to monitor continuously and non-invasively the oxygen and carbon dioxide status of patients with cardio-respiratory disorders. The transcutaneous technique has found special application in newborn infants, whose skin is thinner and has a greater density of capillaries, and in whom transcutaneous $p_{O_2}$ correlates closer to arterial blood $p_{O_2}$ than in adults. The purpose of this document is to provide guidelines for the terminology, methodology, and for the interpretation of data obtained from the use of skin electrodes. Arterial blood gas values are the reference points, and should be measured in connection with transcutaneous measurements. Recommendations covering the technical aspects of transcutaneous electrodes have been prepared by The American Society for Testing and Materials [1] and The International Electrotechnical Commission [2].

### 2. Definitions, terminology and abbreviations

The $p_{O_2}$ (or $p_{CO_2}$) of the heated skin surface is usually used as an indirect (transcutaneous) measure of the $p_{O_2}$ (or $p_{CO_2}$) of arterial blood. In this case, the system of measurement is arterial blood, although the result is modified by the special transcutaneous technique, and the correct symbol in line with IFCC/IUPAC [3] is $p_{O_2(aB,\ tc)}$ (or $p_{CO_2(aB,\ tc)}$), which may be shortened to $p_{O_2(tc)}$ (or $p_{CO_2(tc)}$).

An alternative viewpoint is that the measurement system is the heated skin surface and the quantity is therefore distinct from the $p_{O_2}$ (or $p_{CO_2}$) of arterial blood. In this case, it is more appropriate to use the symbol $p_{O_2(S)}$ (or $p_{CO_2(S)}$), where S is the heated skin surface. Other commonly used symbols for transcutaneous $p_{O_2}$ (or $p_{CO_2}$) are tc$p$O$_2$ (or tc$p$CO$_2$), $p$tcO$_2$ (or $p$tcCO$_2$), PtcO$_2$ (or PtcCO$_2$) and P$_s$O$_2$ (or P$_s$CO$_2$), although these are not in line with IFCC/IUPAC definitions [3].

### 3. Physiological basis for transcutaneous gas measurements

For a comprehensive review the reader is referred to Huch *et al.* [4], as well as the proceedings of the international symposia on continuous blood gas monitoring [5, 6]. The measured transcutaneous values are the result of several variables, which in turn are modified by heating the skin surface [7].

### *3.1. Variables determining $p_{O_2(tc)}$ and $p_{CO_2(tc)}$*

(*a*) Arterial blood $p_{O_2}$ and $p_{CO_2}$ ($p_{O_2(aB)}$ and $p_{CO_2(aB)}$), and, to a lesser extent, the position and shape of the oxygen and carbon dioxide binding curves.

(*b*) Capillary blood flow in the skin under the electrode.

(*c*) Oxygen consumption and carbon dioxide production by the skin.

(*d*) Oxygen consumption by the electrode.

(*e*) Temperature gradients in the skin.

(*f*) Structure and diffusive properties of the skin.

### *3.2. Effects of heating the skin surface*

(*a*) Increase in capillary blood flow in the skin under the electrode, with diminished arterio-venous differences in $p_{O_2}$ and $p_{CO_2}$.

Correspondence should be addressed to: P. D. Wimberley, Department of Clinical Chemistry, Herning Hospital, DK 7400 Herning, Denmark.

† Committee members are: A. H. J. Maas (NL), Chairman; R. W. Burnett (US); A. K. Covington (UK); O. Mueller-Plathe (DE); P. D. Wimberley (DK); and W. G. Zijlstra (NL).

© 1989 IFCC.

(*b*) Lowered blood solubility of $O_2$ and $CO_2$, as well as lowered haemoglobin-oxygen affinity and increased dissociation of carbonic acid. All these factors cause an increase in $p_{O_2}$ and $p_{CO_2}$ in the heated blood.

(*c*) Increased diffusion of gases through the skin due to solubilization of the lipid layer of the epidermis.

(*d*) Increased $O_2$ consumption and $CO_2$ production by the skin.

From this it follows that the relation between $p_{O_2(aB)}$ and $p_{CO_2(tc)}$ is complex, as is that between $p_{CO_2(aB)}$ and $p_{CO_2(tc)}$ (see section 8.2).

### 4. Instrumentation and equipment

### *4.1. The electrode system*

Transcutaneous $p_{O_2}$ electrodes are heated Clark-type electrodes, and transcutaneous $p_{CO_2}$ electrodes are heated Stow-Severinghaus electrodes. The two sensors may be combined in a single housing with a common electrolyte solution, membrane, and Ag/Cl reference electrode. The system contains a thermostatically controlled heating element with preset temperatures ranging from 37·0 to 45·0 °C. Although not presently available, optical sensors for the measurement of $p_{O_2(tc)}$ and $p_{CO_2(tc)}$ are under development [8, 9].

### *4.2. The monitor*

The monitor should include:

(*a*) Circuitry for $p_{O_2}$ and $p_{CO_2}$ measurement.

(*b*) Heating and temperature control from 37·0 to 45·0 °C.

(*c*) Preferably a digital display, and outputs for recorder and for printer of $p_{O_2}$, $p_{CO_2}$ and heat consumption by the heating element.

(*d*) Calibration adjustment controls.

(*e*) Means of testing the electrical resistance of the membrane.

(*f*) Alarm (visual and auditory) which activates if (1) $p_{O_2(tc)}$ or $p_{CO_2(tc)}$ are outside pre-set limits selected by the clinician, or (2) electrode temperature deviates by more than 0·3 °C from the selected temperature.

### 5. Calibration

Calibration should be performed at the electrode measuring temperature immediately prior to use and before each re-application of the electrode to the patient (i.e. normally every 4 h or less). Any fluid droplets on the outer side of the electrode membrane should be carefully removed before calibration. Calibration gas mixtures should have a relative inaccuracy of less than ±0·5% of the stated value. The composition of the gas mixtures does not change with storage.

### *5.1. $p_{O_2(tc)}$ electrode*

Zero adjustment is usually performed automatically, assuming zero current at zero $p_{O_2}$, but may be checked with a 'zero' solution containing an oxygen consuming agent, such as sodium sulphite or with $N_2$ gas.

Calibration is generally made with atmospheric air, or with a certified gas mixture containing known fractions of oxygen and carbon dioxide, balanced with nitrogen.

The $p_{O_2}$ of atmospheric air is:

$$p_{O_2(atm)} = 0{\cdot}2093 \times (p(atm) - p_{H_2O(atm)})$$

where 0·2093 is the volume fraction of $O_2$ in atmospheric air, $p$(atm) is the ambient pressure, $p_{H_2O(atm)}$ is the partial pressure of $H_2O$ in the air, and may be calculated from the relative humidity ($r_{H_2O}$) and the saturated water vapour pressure at the ambient temperature ($p_{H_2O}$).

*For example:* if $p(atm) = 100$ kPa, $p_{H_2O} = 2{\cdot}6$ kPa, $r_{H_2O} = 0{\cdot}4$, then $p_{O_2(atm)} = 0{\cdot}2093 \times (100 - (2{\cdot}6 \times 0{\cdot}4))$ kPa = 20·7 kPa.

The electrode sensitivity (d Current/d$p_{O_2}$) should be within the manufacturer's specified limits, which depend on the cathode size and the membrane permeability and thickness. Furthermore, a stable calibration value should be attained within 3 min. Failure to satisfy these requirements should be indicated by an error message.

### *5.2. $p_{CO_2(tc)}$ electrode*

Two-point calibration is performed at least once a day with two dry gas mixtures with known $CO_2$ fractions, for example 0·05 and 0·10 ($p_{CO_2}$ about 5 and 10 kPa respectively), with a one-point calibration (for example, with $F_{CO_2(G)} = 0{\cdot}05$) more frequently.

The $p_{CO_2}$ of a dry gas is:

$$p_{CO_2(G)} = F_{CO_2(G)} \times p(atm).$$

For example, if $p(atm) = 100$ kPa and $F_{O_2(G)} = 0{\cdot}05$, $p_{CO_2(G)} = 0{\cdot}05 \times 100$ kPa = 5·0 kPa.

The electrode sensitivity ($dE/d\lg p_{CO_2}$) should be within 5% of the theoretical.

### 6. Measurement procedure

A typical protocol is described, details of which may vary according to the electrode system and monitor used. Detailed instructions should be found in the manuals produced by the manufacturers.

### *6.1. Selection of measurement site*

Optimal measuring conditions are obtained in skin areas with high density of capillaries, ample capillary blood flow, thin epidermis, and small or no deposits of fat. The optimal places are the lateral sides of the abdomen and chest. The arms and legs are best avoided, as vasoconstriction, reducing skin blood flow, occurs earlier here if the patient becomes cold or hypotensive. In those newborn infants where there is a possibility of right-to-left shunting through the *ductus arteriosus*, the electrodes must be placed on the right upper chest to detect/avoid high $p_{O_2}$ levels in the blood supplying the retina.

### 6.2. *Preparation of skin*

Hair should be removed to ensure better adhesion. Cleaning the skin with alcohol is recommended.

### 6.3. *Fixation of the electrode*

Fixation to the skin is facilitated by a self-adhesive ring.

### 6.4. *Contact liquid*

Contact between the electrode and the skin is best ensured by a thin layer of fluid (for example, water or glycerol). It is important to avoid the presence of air between the electrode and the skin, and not to moisten the adhesive tape.

### 6.5. *Selection of electrode temperature*

$p_{O_2(tc)}$ is usually measured at 44 °C, and $p_{CO_2(tc)}$ at 42 °C (except in the combined $p_{O_2}/p_{CO_2}$ sensor where $p_{CO_2(tc)}$ is also measured at 44 °C). The higher temperature is necessary for $p_{O_2(tc)}$ because of the poorer diffusion of $O_2$ through the skin, compared to $CO_2$, and because of the greater arteriovenous differences for $p_{O_2}$ compared to $p_{CO_2}$. Serious skin burns may occur with electrode temperatures over 44 °C, even with measuring times under 4 h (see section 7.1).

### 6.6. *Comparison with arterial blood values*

In order to ensure the correct interpretation of transcutaneous values, at the start of each patient monitoring period, the relationship between $p_{O_2(tc)}$ and $p_{O_2(aB)}$, and between $p_{CO_2(tc)}$ and $p_{CO_2(aB)}$, should be established. Furthermore, it should be remembered that any change in $p_{O_2(tc)}$ may be due to either a change in $p_{O_2(aB)}$, or due to a change in skin blood flow (see section 8.2.1). $p_{O_2(tc)}$ and $p_{CO_2(tc)}$ only reflect $p_{O_2(aB)}$ and $p_{CO_2(aB)}$, respectively, at relatively high local blood flow.

Capillary blood cannot replace arterial blood as the reference system because the presence of even very small amounts of venous blood, which commonly contaminate the specimen, may result in a marked decrease in capillary blood $p_{O_2}$ compared to arterial blood $p_{O_2}$.

### 6.7. *Measurement time*

(See section 7.1.)

### 6.8. *Electrode drift check*

After removal from the skin, the electrode reading should be checked in a calibration gas, in order to document any electrode drift which may have occurred during measurement on the skin (see section 5). This should be less than ±5% for both $p_{O_2(tc)}$ and $p_{CO_2(tc)}$ over the maximum recommended calibration period of 4 h. The gas should have the same temperature and flow rate as that used for calibration.

### 6.9. *Use of recorder*

A recorder is recommended because it has the advantage of providing retrospective analysis of changes in $p_{O_2(tc)}$ and $p_{CO_2(tc)}$. A recorder, however, increases the size of the monitoring system, and reduces its portability. Trends may also be observed by a visual display unit.

### 7. Patient safety

### 7.1. *Avoidance of skin burns*

Prolonged application of a heated transcutaneous electrode may cause skin burns. For this reason an electrode temperature above 44 °C is not recommended for routine clinical monitoring.

At an electrode temperature of 44 °C, the electrode measuring site must be changed at least every 4 h, in order to avoid blister formation. Even after shorter measuring times, the skin will show erythema when the electrode is removed, though this erythema disappears after one or two days. The risk of skin burning is increased in patients with peripheral circulatory failure, and in very small, premature infants and therefore the measuring site should be changed more frequently, for example every 2–3 h. An alarm should be included to ensure that the device automatically turns off within a few seconds, should the electrode core temperature rise above 46 °C.

Two thermistors are recommended to ensure back up in the event of failure.

See also section 9.1 on electrode temperature control.

### 7.2. *Electrical safety*

All equipment used should be certified by its manufacturer to comply with a national or international electrical safety standard. The equipment must not interfere electrically with other equipment (for example an electrocardiogram or a heart pacemaker) attached to the patient.

### 8. Performance characteristics

### 8.1. *Imprecision*

Imprecision may be due to the electrode drift, variations in pressure on the electrode, and the variable properties of the skin. Coefficients of variation (CV) determined from duplicate measurements in the same individual may be expected to be about 10% for $p_{O_2(tc)}$ and 5% for $p_{CO_2(tc)}$ [10, 11]. These figures include both biological and analytical variation. The same electrode should have a CV of only 1–2%, when duplicate measurements are made in dry gases [10].

*8.2. Accuracy*

*8.2.1. Bias in* $p_{O_2(tc)}$: $p_{O_2(tc)}$ values at 44 °C are approximately equal to $p_{O_2(aB)}$ values at 37 °C in newborn infants [4, 12, 13]. However, large biases in $p_{O_2(tc)}$ are common [10], especially in adults [7, 14] due to the following causes:

(*a*) Variation in the $p_{O_2}$ temperature coefficient with increasing $p_{O_2}$ level, gives a negative bias in $p_{O_2(tc)}$ at high $p_{O_2}$ levels, i.e. $p_{O_2(tc)}$ reads too low. Other factors, for example abnormal total haemoglobin concentration and an altered haemoglobin oxygen affinity, also give a bias, but these effects are smaller [15].

(*b*) Variation in skin capillary blood flow: decrease in skin capillary blood flow causes a decrease in $p_{O_2(tc)}$. Changes in blood flow may be due to sepsis, changes in blood $p_{CO_2}$, administration of drugs, for example tolazoline and catecholamines, and furthermore may be general or localized to the area of skin under the electrode.

(*c*) Variations in skin anatomy: increasing skin thickness and decreasing skin vascularization, which, for example occur with increasing age, result in low $p_{O_2(tc)}$ values. Differences in skin anatomy between various parts of the body also cause bias.

(*d*) Interference from anaesthetic gases: halothane and nitrous oxide may give falsely elevated $p_{O_2(tc)}$ values [16–19].

Due to the large variations in the degree of bias, $p_{O_2(tc)}$ cannot be used to replace $p_{O_2(aB)}$. The major use of $p_{O_2(tc)}$ is as a trend parameter for continuous non-invasive monitoring, using $p_{O_2(aB)}$ as the reference point.

However, in adults, studies have shown that $p_{O_2(tc)}$ is often not a trend indicator of $p_{O_2(aB)}$, but may be a valuable indicator of tissue $p_{O_2}$ in preterminal and terminal patients during periods of hypoxic shock and cardiac arrest [20–22].

*8.2.2. Bias in* $p_{CO_2(tc)}$: $p_{CO_2(tc)}$ values at 42–45 °C are higher than $p_{CO_2(aB)}$ at 37 °C. However, the relative bias is much more constant than that for $p_{CO_2(tc)}$ [10, 11], such that when corrected to body temperature, $p_{CO_2(tc)}$ provides a better indicator of $p_{CO_2(aB)}$ than can be said for $p_{O_2(tc)}$ and $p_{O_2(aB)}$. Several methods of 'correcting' $p_{CO_2(tc)}$ to estimate $p_{CO_2(aB)}$ have been used, but none have been sufficiently well documented to be considered as standard. A single factor is known to be insufficient in that, when normal values are correctly obtained, the resulting slope of the electrode is low for changes from normal, underestimating the degree of abnormality in either direction. An empiric 'skin metabolism offset' of about 0·8 kPa used in connection with a temperature coefficient, $\mathrm{dln}p_{CO_2}/\mathrm{d}\theta = 0{\cdot}04 \times {}^\circ\mathrm{C}^{-1}$ (corresponding to about 4% per °C), has been shown to provide reasonable correction and tracking of variation of $p_{CO_2}$ values, i.e. the $p_{CO_2(tc)}$ value measured at an electrode temperature $\theta(\mathrm{E})$ is divided by exp $(0{\cdot}04\ ((\theta(\mathrm{E})/{}^\circ\mathrm{C}) - 37))$ and then reduced by 0·8 kPa. The temperature coefficient of 0·04 used here is slightly lower than the anaerobic temperature coefficient for $p_{CO_2}$ in blood ($\mathrm{dln}p_{CO_2}/\mathrm{d}\theta = 0{\cdot}048 \cdot {}^\circ\mathrm{C}^{-1}$ [23]) because the skin capillary temperature is slightly lower than the electrode temperature [24, 25].

### 9. Quality assurance

*9.1. Electrode temperature control*

Temperature control should be accurate to within 0·3 °C. This should be checked periodically by placing the electrode in a temperature-controlled environment. With the heater power off, the electrode monitor's temperature measurement should be within ±0·3 °C of the environmental temperature [1]. With heater power on, and monitor temperature set to the environmental temperature, the monitor heat output should read zero.

### 9.2. Electrode drift rate

When placed in a calibration gas, $p_{O_2}$ and $p_{CO_2}$ should not drift more than ±1% per hour. For drift during patient monitoring, see section 6.8.

### 9.3. Changes in heat consumption during patient monitoring

A decrease in $p_{O_2(tc)}$ value, together with decreased heat dissipated by the heating element, indicates reduced blood flow in the skin capillaries and in the deeper and larger vessels under the electrode, and not necessarily reduction in $p_{O_2(aB)}$. However, changes in heat consumption also occur with changes in the environmental and the patient's temperature.

### Acknowledgements

Constructive comments on this document have been received from J. W. Severinghaus, D. W. Lübbers, E. O. R. Reynolds, A. and R. Huch, I. Fatt, D. Delpy, I. H. Göthgen, E. Jacobsen, AACC Electrolyte/Blood Gas Division, NCCLS, The Association of Clinical Biochemistry, The Australian Association of Clinical Biochemistry, The Italian Society of Clinical Biochemistry, Center for Devices and Radiological Health, Radiometer, Corning, and Instrumentation Laboratories.

### References

1. American Society for Testing and Materials Committee. *Cutaneous Gas Monitoring Devices for Oxygen and Carbon Dioxide, Standard Specification, F 984–86* (American National Standards Institute, 1430 Broadway, New York, New York 10017, USA, 1986).
2. International Electrotechnical Commission. *Particular Requirements for the Performance of Transcutaneous Oxygen and Carbon Dioxide Partial Pressure Monitoring Equipment* (IEC Technical Committee 62D, Draft Publication 601–3, 1987).

3. Siggaard-Andersen, O., Durst, R. A. and Mass, A. H. J., *Journal of Clinical Chemistry and Clinical Biochemistry,* **25** (1987), 369.
4. Huch, R., Huch, A. and Lübbers, D. W., *Transcutaneous $PO_2$* (Thieme-Stratton Inc., New York, 1981).
5. Huch, A., Huch, R. and Lucey, J. F. (Eds), *Continuous Transcutaneous Blood Gas Monitoring. Birth Defects,* Original Article Series Volume 15, No. 4 (Alan R. Liss Inc., New York, 1979).
6. Huch, R. and Huch, A. (Eds), *Continuous Transcutaneous Blood Gas Monitoring* (Marcel-Dekker Inc., New York, 1983).
7. Grønlund, J., *Journal of Applied Physiology,* **59** (1985), 1117.
8. Lübbers, D. W. and Opitz, N., in *Proceedings of the International Meeting on Chemical Sensors, Fukuova* (Elsevier, The Netherlands, 1983).
9. Gehrich, J. L., Lübbers, D. W., Opitz, N. *et al., IEEE Transactions on Biomedical Engineering,* **2** (1986), 117.
10. Wimberley, P. D., Frederiksen, P. S., Witt-Hansen, J., Melberg, S. G. and Friis-Hansen, B., *Acta Pediatr. Scand.,* **74** (1985), 352.
11. Wimberley, P. D., Grønlund Pederson, K., Olsson, J. and Siggaard-Andersen, O., *Clinical Chemistry,* **31** (1985), 1611.
12. Eberhard, P., *Continuous Oxygen Monitoring of Newborns by Skin Sensors,* Thesis (Offset Press, Basel, 1976).
13. Löfgren, O., *On Transcutaneous $PO_2$ Measurement in Humans. Some Methodological, Physiological and Clinical Studies,* Thesis (Malmö, 1978).
14. Lübbers, D. W. and Grossmann, U.,. in *Continuous Transcutaneous Blood Gas Monitoring,* Eds Huch, R. and Huch, A. (Marcel-Dekker Inc., New York, 1983), p. 1.
15. Siggaard-Andersen, O., Wimberley, P. D., Gøthgen, I. and Siggaard-Andersen, M., *Clinical Chemistry,* **30** (1984), 1646.
16. Severinghaus, J. W., Weiskopf, R. B., Nishimura, M. and Bradley, A. F., *Journal of Applied Physiology,* **31** (1971), 640.
17. Dent, J. G. and Netter, K. J., *British Journal of Anaesthesia,* **48** (1976), 195.
18. Albery, W. J., Brooks, W. N., Gibson, S. P. and Hahn, C. E. W., *Journal of Applied Physiology,* **45** (1978), 637.
19. Eberhard, P. and Mindt, W., in *Continuous Transcutaneous Blood Gas Monitoring, Birth Defects,* Original Article Series Volume 15, No. 4, Eds Huch, A., Huch, R. and Lucey, J. F. (Alan R. Liss Inc., New York, 1979), p. 65.
20. Tremper, K. K., Waxman, K., Bowman, R. and Shoemaker, W. C., *Critical Care Medicine,* **8** (1980), 377.
21. Nolan, L. S. and Shoemaker, W. C., *Critical Care Medicine,* **10** (1982), 762.
22. Shoemaker, W. C. and Tremper, K. K., in *Continuous Transcutaneous Blood Gas Monitoring,* Eds Huch, R. and Huch, A. (Marcel-Dekker Inc., New York, 1983), p.745.
23. Siggaard-Andersen, O. *The Acid-Base Status of the Blood,* 4th edn. (Munksgaard, Copenhagen, 1974), p. 89.
24. Severinghaus, J. W., *Respiratory Care,* **27** (1982), 152.
25. Severinghaus, J. W. and Naifeh, K. H., *Journal of Applied Physiology,* **64** (1968), 391.

### Addendum

Since the approval of this document by IFCC, there has been an increasing interest in the possibility of using transcutaneous $p_{O_2}$ and $p_{CO_2}$ on human foetal scalp during labour as an additional parameter to detect foetal asphyxia. Although several authors advocate this technique [1, 2], the technical problems, and the importance of a simultaneous scalp blood flow measurement [3], necessarily limit this application to research purposes at present.

### References

1. Nickelsen, C., *Danish Medical Bulletin,* **36** (1989), 537.
2. Schmidt, S. C. and Saling, E. Z., *British Journal of Obstetrics and Gynaecology,* **94** (1987), 963.
3. Smits, T. M., Aarnouds, J. G. and Zijlstra, W. G., *Early Human Development,* **20** (1989), 109.

